# Development of an immunogenomic landscape for the competing endogenous RNAs network of peri-implantitis

**DOI:** 10.1186/s12881-020-01145-4

**Published:** 2020-10-20

**Authors:** Yang Li, Jina Zheng, Chanjuan Gong, Kengfu Lan, Yuqing Shen, Xiaojun Ding

**Affiliations:** 1 Department of Stomatology, Zhongshan Hospital, Fudan University, No. 180 Fenglin Road, Xuhui District, Shanghai, 200032 China; 2 State key laboratory of molecular engineering of polymers, Fudan University, Shanghai, P.R. China; 3 Department of Dermatology, Zhongshan Hospital, Fudan University, Shanghai, P.R. China

**Keywords:** Competing endogenous RNAs (ceRNA), Peri-implantitis, Immune microenvironment, GSK3B, miR-1297

## Abstract

**Background:**

Peri-implantitis is an inflammation that occurs around the implant, resulting in varying degrees of inflammatory damage to the soft and hard tissues. The characteristic criterion is the loss of the supporting bone in an inflammatory environment. However, the specific mechanisms and biomarkers involved in peri-implantitis remain to be further studied. Recently, competing endogenous RNAs (ceRNA) and immune microenvironment have been found to play a more important role in the inflammatory process. In our study, we analyzed the expression of immune related microRNAs (miRNAs), long noncoding RNAs (lncRNAs) and message RNAs (mRNAs) in peri-implantitis by analyzing GSE33774 and GSE57631.

**Methods:**

In this study, we explored the expression profile data of immune-related lncRNAs, miRNAs and mRNAs, and constructed immune-related ceRNA network involved in the pathogenesis of peri-implantitis. In addition, the CIBERSORT was used to evaluate the content of immune cells in normal tissues and peri-implantitis to detect the immune microenvironment of peri-implantitis.

**Results:**

In the analysis, 14 DElncRNAs, 16 DEmiRNAs, and 18 DEmRNAs were used to establish an immune related ceRNA network and the immune infiltration patterns associated with peri-implantitis was discovered. Through the mutual verification of the two datasets, we found that GSK3B and miR-1297 may have important significance in the immune microenvironment and pathogenesis of peri-implantitis and GSK3B was closely related to four types of immune cells, especially with the highest correlation with resting mast cells (*P* = 0.0003).

**Conclusions:**

Through immune-related ceRNA network, immune-related genes (IRGs) and immune cell infiltration can further comprehensively understand the pathogenesis of peri-implantitis, which built up an immunogenomic landscape with clinical significance for peri-implantitis.

## One-sentences summary

GSK3B and miR-1297 may have important significance in the immune microenvironment and pathogenesis of peri-implantitis.

## Background

With the advantages of stability, low foreign body sensation, non-injury to adjacent teeth and high masticatory efficiency, dental implants have become an important means to repair dentition defect or absence, and have been accepted by the general public. Peri-implant diseases include peri-implant mucositis and peri-implantitis [1]. Mucositis is defined as a bacteria-induced, reversible inflammatory process of the peri-implant soft tissue with reddening, swelling and bleeding on periodontal probing, and peri-implantitis as a bacteria-induced, progressive and irreversible disease of implant-surrounding hard and soft tissues and is accompanied with bone resorption, decreased osseointegration, increased pocket formation and purulence [2].

Currently, the incidence of peri-implantitis is high and can lead to failure of the implant, wherein the plaque is the initiating factor. There is no periodontal membrane tissue support around the implant, and there are fewer peripheral blood vessels and fibroblasts. Oral soft tissue surrounds the perforated gingival part of the implant, forming epithelial cuff of the implant, thus forming a protective barrier around the implant. However, due to the special structure of the epithelial cuff, its defense ability weakened, and bacteria are easy to penetrate. Once bacteria break through the epithelial cuff, they can quickly colonize the bone tissue, causing the damage to the soft and hard tissue surrounding the implant, and forming inflammation of the tissue around the implant [3].

The immune response refers to the physiological process produced by the body’s immune system to stimulate antibody for the purpose of eliminating antigens. Peri-implantitis and periodontitis were similar in the etiology and pathogenesis. Similar to periodontitis, bacterial invasion of the surrounding tissue can trigger an immune response. On the one hand, the reaction can remove harmful substances such as bacteria and toxins; on the other hand, the cytokines, proteases and prostaglandins produced in the process will accelerate the destruction of the tissues around the implant [4]. With the increasing popularity of dental implants, peri-implantitis has attracted great attention, but its etiology and pathogenesis are still unclear. Immune response is the key to explore the pathogenesis of peri-implantitis.

Competing endogenous RNAs (ceRNA) are a type of non-coding RNAs that can bind miRNA response elements (MREs) to inhibit the formation of silencing complexes (miRISCs) and further increase the expression of corresponding mRNA to achieve mutual communication between RNAs and regulate gene expression after transcription [5]. CeRNA mainly includes microRNAs (miRNAs), long noncoding RNAs (lncRNAs), circular RNAs (circRNAs), pseudogenes, synthetic miRNA inhibitors and viral miRNA inhibitors. Among them, miRNAs and lncRNAs are the most important types. LncRNAs, miRNAs, and mRNAs interact with each other to form regulatory competitive endogenous RNA networks [6]. Recent studies have shown that there are significant differences in the expression profile of miRNAs and lncRNAs between inflammatory and healthy gingival tissue in patients with periodontitis [7]. However, the ceRNA network involved in the pathogenesis of peri-implantitis has not been reported by scholars. In this study, we explored the expression profile data of immune-related lncRNAs, miRNAs and mRNAs, and constructed immune-related ceRNA network involved in the pathogenesis of peri-implantitis, so as to further discover the key immune-related genes (IRGs) in peri-implantitis. In addition, we also studied the phenomenon of immune cell infiltration in peri-implantitis. Through immune-related ceRNA network, IRGs and immune cell infiltration can further comprehensively understand the pathogenesis of peri-implantitis, which also provides a new perspective for the diagnosis and treatment target of peri-implantitis. In conclusion, our study has built up an immunogenomic landscape with clinical significance for peri-implantitis.

## Methods

### Datasets and data processing

The gene expression data of peri-implantitis patients from GSE33774 (https://www.ncbi.nlm.nih.gov/geo/query/acc.cgi?acc=GSE33774) were downloaded from the Gene Expression Omnibus (GEO). In total, we downloaded the raw data of 7 peri-implantitis and 8 normal tissues for analysis and used the RMA method to preprocess the original data. The IRGs were downloaded from the ImmPort database (https://www.immport.org/), which provides an accurate and up-to-date list of IRGs that participating in immunization activities [8].

### Differentially expressed genes analysis

The “Limma” package of R software (version: × 64 3.6.1) were used to selected and differentially expressed lncRNAs (DElncRNAs) and differentially expressed genes (DEGs) involved in peri-implantitis [9]. In addition, |log (FC)| > 1 and false discovery rate (FDR) < 0.05 were defined as the screening criteria for DEGs and DElncRNAs. The expression of all IRGs were obtained from DEGs. Heatmaps of IRGs and DElncRNAs were constructed, using the “Pheatmap” package.

### Exploration of the ceRNA network

The lncRNA-miRNA interactions were determined based on data from the miRcode database (http://www.mircode.org/) [10]. Then to predict the miRNA and mRNA interaction, the database of miRDB (http://mirdb.org/), TargetScan (http://www.targetscan.org/vert_72/) and miRTarBase (http://mirtarbase.mbc.nctu.edu.tw/php/index.php) were utilized. The obtained target genes were intersected with IRGs [11–13]. The Cytoscape software 3.7.1 was used to visualized the lncRNA-miRNA-mRNA network with the interactions of results [14].

### Function and pathway enrichment

The Gene Ontology (GO) function and Kyoto Encyclopedia of Genes and Genomes (KEGG) pathway enrichment analysis for the IRGs in ceRNA network were analyzed by using the “clusterProfiler” package of R software, and *P* value < 0.05 was considered statistically significant [15].

### Validation of ceRNA network in another GEO dateset

In order to further verify the accuracy of immune-related lncRNAs, miRNAs and mRNAs obtained, we verified it again in another dataset. We downloaded the gene expression of 2 normal tissue and 6 peri-implantitis from GSE57631 (https://www.ncbi.nlm.nih.gov/geo/query/acc.cgi?acc=GSE57631) and constructed the ceRNA network in the same way. The results showed that only GSK3B and miR-1297 existed in both ceRNA networks.

### Validation of GSK3B by Weighted Gene Co-expression Network Analysis (WGCNA)

Then we combined GSE33774 and GSE57631 and normalized them. Finally, we obtained the gene expression profiles of 10 normal tissues and 13 peri-implantitis. In order to validate GSK3B whether associated with peri-implantitis, we selected the top 25% genes with the largest variance as the input database and used the “WGCNA” package of R software (version: × 64 3.6.1) to construct a weighted gene network. The standard scale-free network is constructed by selecting the soft threshold of the appropriate adjacency matrix. Next, we transformed the adjacency matrix into topological overlap matrix (TOM), used hierarchical clustering to generate the clustering gene dendrogram and set the minimum module size to 30. After determining the gene modules by dynamic tree cut method, we calculated the eigengenes of each module in turn, and then conducted cluster analysis to merge the modules that were close to each other into a new module (height = 0.25). The merged dynamic was the resulting modules. Next, we analyzed the correlation between modules and traits, selected modules containing GSK3B for further analysis, and obtained gene significance (GS) and module membership (MM) [16].

### Abundance calculations

Immune cell abundance was calculated from the CIBERSORT results. CIBERSORT, a deconvolution algorithm developed by Bindea G et al., can estimate the cell composition of complex tissues based on standardized gene expression data, which can quantify the abundance of specific cell types [17]. CIBERSORT can enumerate 22 immune cell types at once and quantify the relative proportions of each cell type using signatures from 500 marker genes.

### Statistical analysis

In order to verify the relationship between the abundance of these 22 immune cells and the gene expression of glycogen synthase kinase 3B (GSK3B), we calculated the Pearson correlation between them, in which *P* value less than 0.05 was significant.

## Results

### Identification of differentially expressed IRGs and lncRNAs in peri-implantitis

**Fig. 1:**
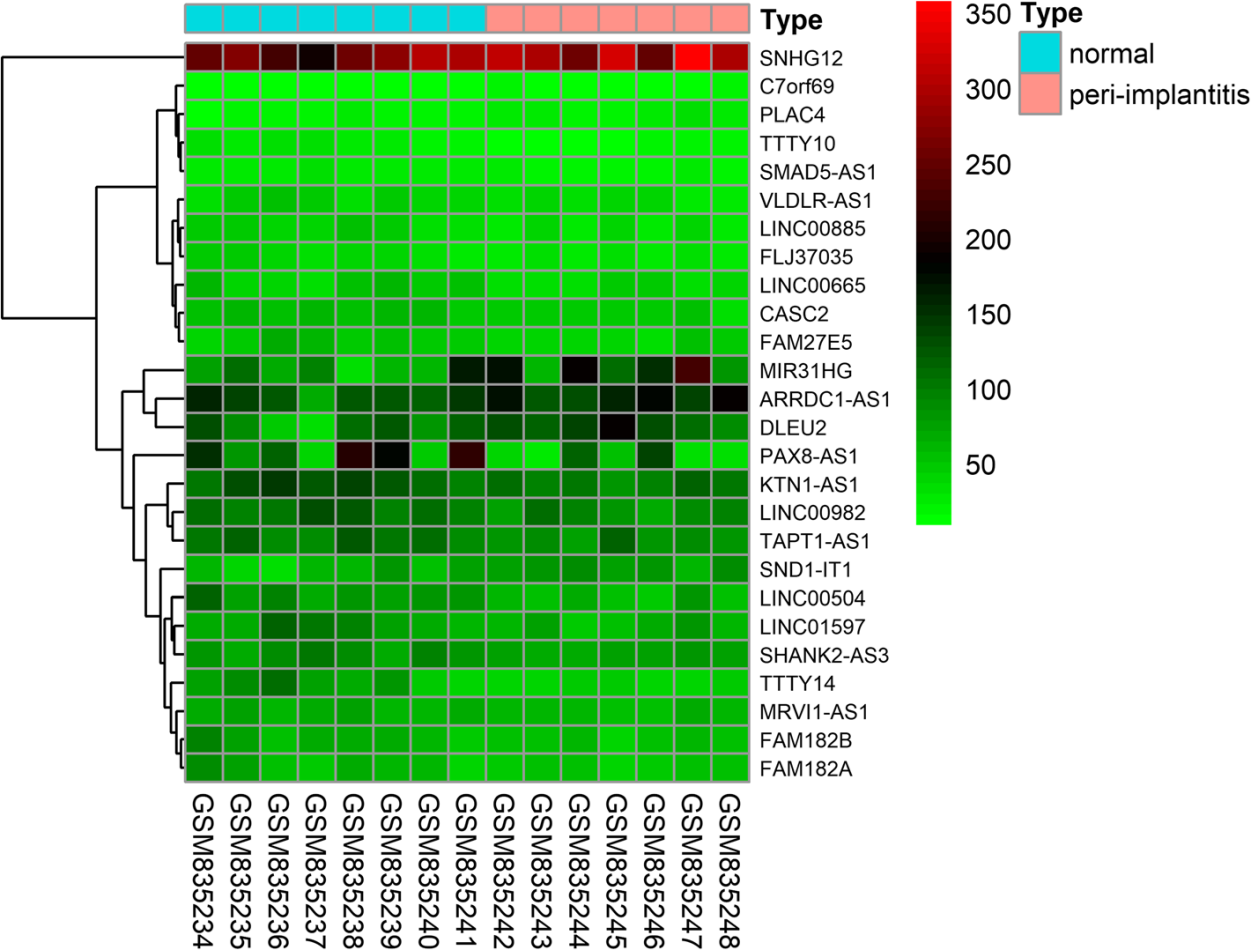
Differentially expressed lncRNAs between peri-implantitis and normal tissues

Using a cut-of threshold of |log2 FC| > 1 and FDR < 0.05, we identified 272 differentially expressed IRGs and 26 DElncRNAs. The heatmap of the DElncRNAs and differentially expressed IRGs showed that the peri-implantitis clustered separately from the normal tissues (Fig. 1 and Figure S1).

### Construction of a ceRNA network for peri-implantitis

**Fig. 2:**
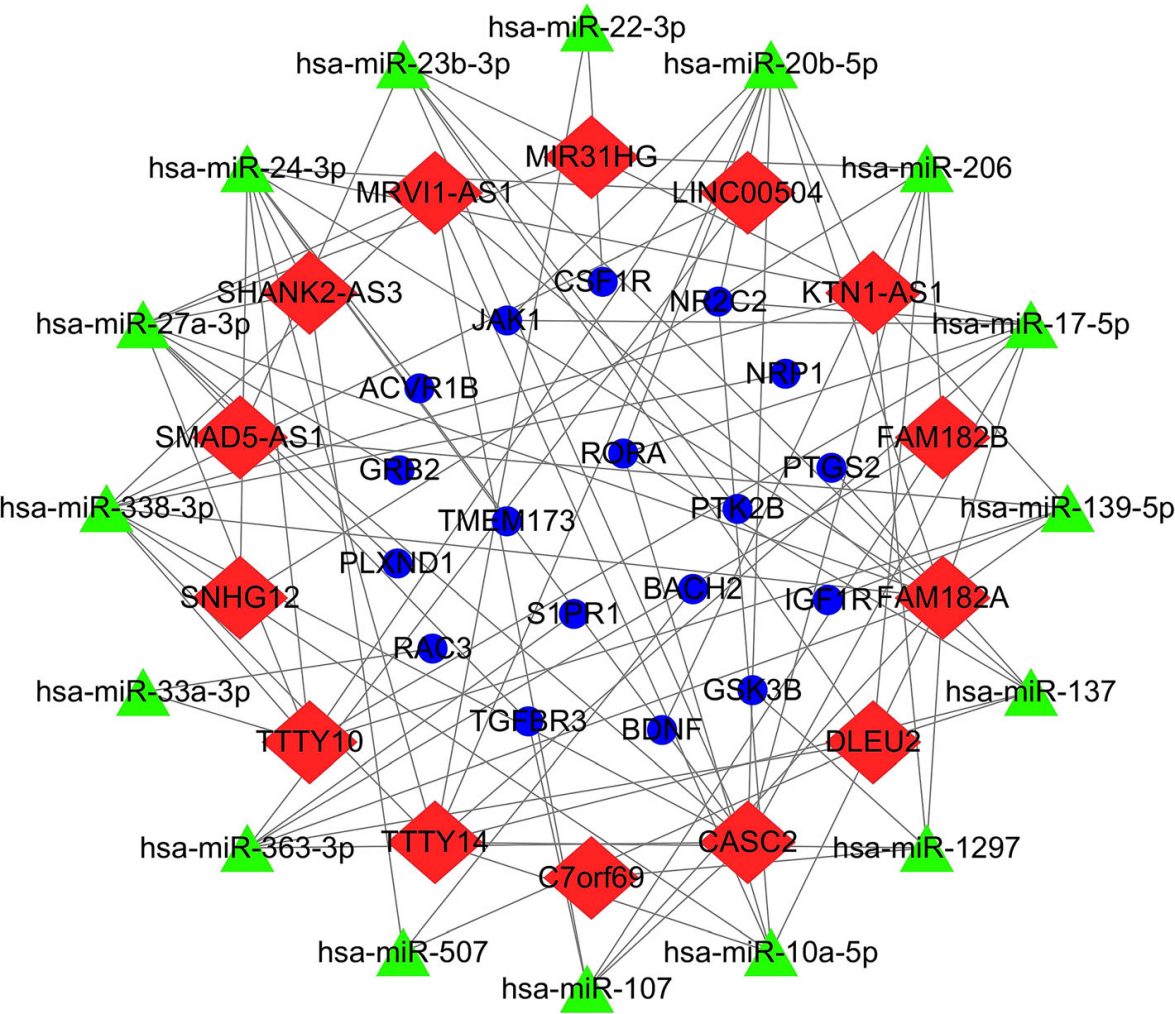
Map of the immune-related lncRNA-miRNA-mRNA network generated using Cytoscape 3.7.1 software

We constructed and visualized the ceRNA network to better understand the role of lncRNAs on mRNAs mediated by combination with miRNAs in peri-implantitis. Through the miRcode database, we retrieved 14 DElncRNAs interacting with 203 DEmiRNAs (Table S1). We then searched for differentially expressed mRNAs (DEmRNAs) from 203 DEmiRNAs in the miRDB, TargetScan and miRTarBase databases. The DEmRNAs we obtained intersected with the previous IRGs, and finally we obtained 14 DElncRNAs, 16 DEmiRNAs and 18 DEmRNAs interacting with each other and established the corresponding ceRNA network (Fig. 2).

### GO enrichment and KEGG signaling pathway analysis of IRGs in ceRNA network

**Fig. 3:**
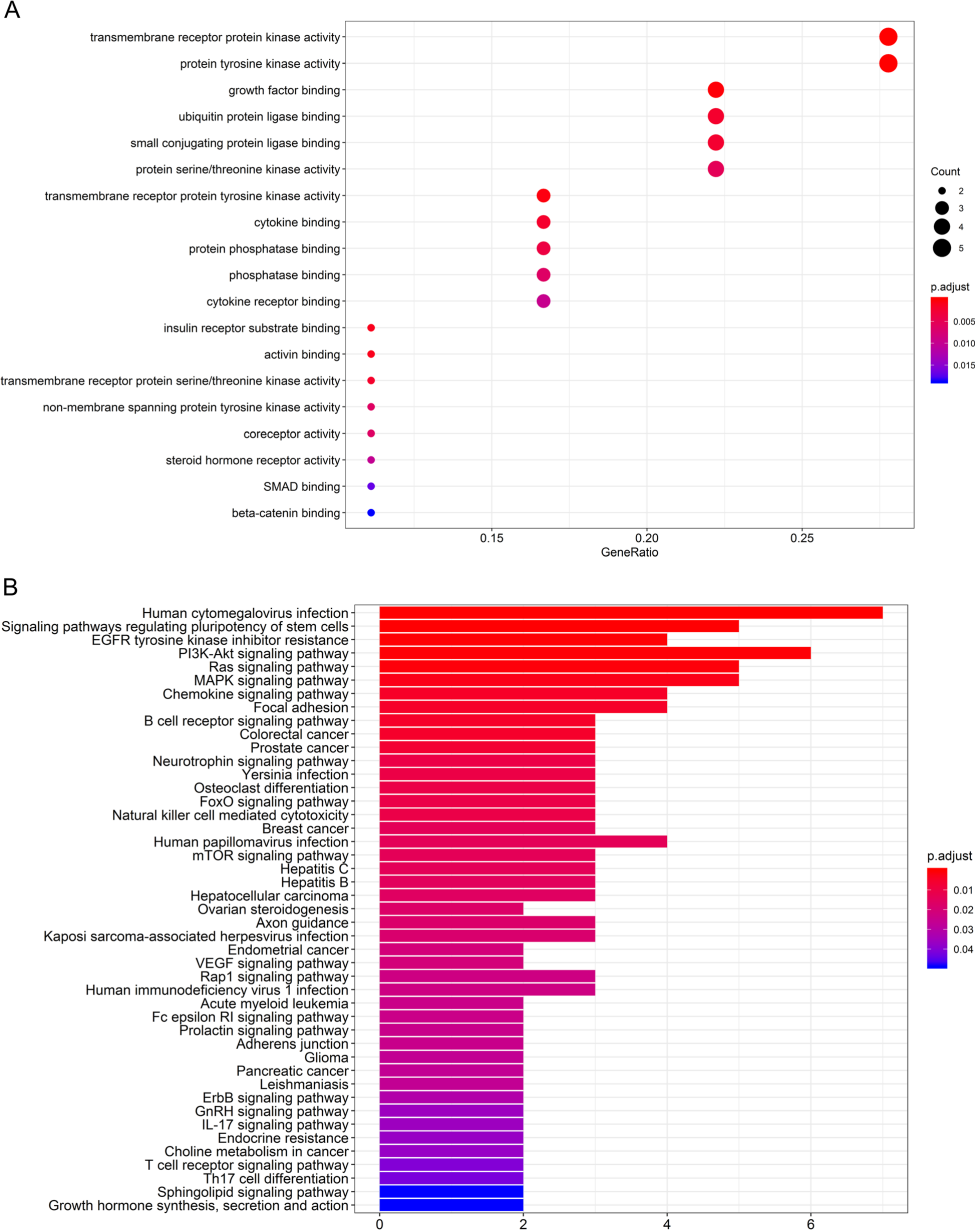
Functional enrichment of differentially expressed immune-related genes (IRGs). **a** The Gene Ontology (GO) annotations of differentially expressed IRGs. **b** The Kyoto Encyclopedia of Genes and Genomes (KEGG) pathways

The GO enrichment analysis is divided into three parts: biological process (BP), cell composition (CC) and molecular function (MF). After screening, it was found that the IRGs in ceRNA network were enriched in transmembrane receptor protein kinase activity, protein tyrosine kinase activity and growth factor binding, suggesting that these IRGs were enriched in inflammatory pathways (Fig. 3a). The KEGG analysis showed that the IRGs were mainly concentrated in human cytomegalovirus infection, MAPK signaling pathway and PI3K-Akt signaling pathway (Fig. 3b).

### Validation of gene expression of differentially expressed IRGs

**Fig. 4:**
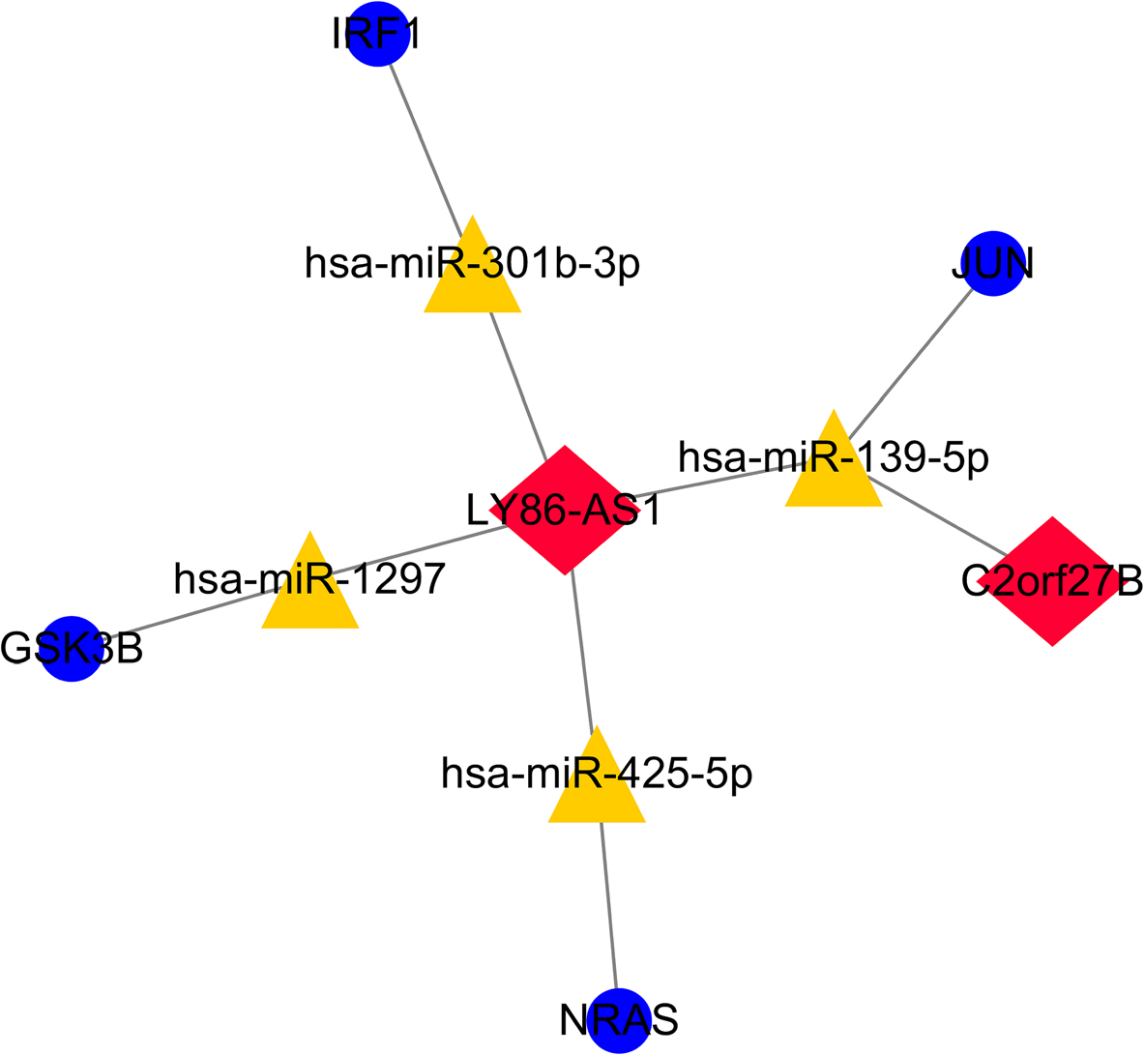
Map of the immune-related lncRNA-miRNA-mRNA network in another dataset GSE57631

By constructing ceRNA network in another dataset GSE57631 in the same way, we found that only GSK3B and miR-1297 existed simultaneously in these two networks. Then we further identified the immune gene GSK3B using the other methods (Fig. 4).

### Co-expression analysis by weighted gene co-expression network analysis and identification of key modules

**Fig. 5:**
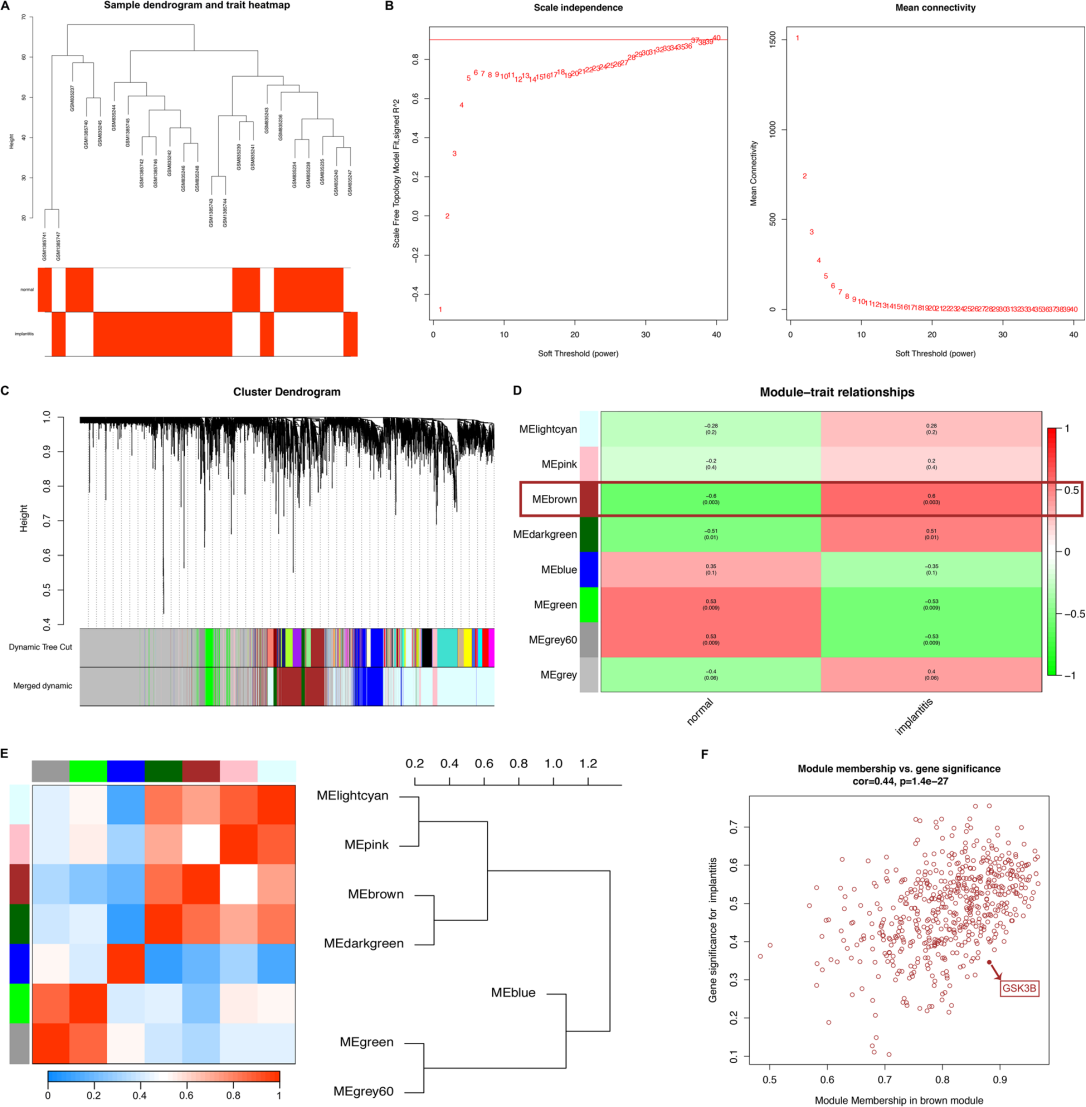
Weighted gene co-expression network construction and identification of the brown module containing GSK3B. **a** Clustering dendogram of samples. **b** The scale independence and the mean connectivity of the weighted gene co-expression network analysis (WGCNA) analysis of peri-implantitis. Testing the scale free topology when β = 32. **c** Clustering dendrograms and modules identified by WGCNA. **d** Correlation between module eigengenes and clinical traits. The brown module containing GSK3B is selected. **e** The cluster tree and eigengene adjacency heatmap between modules. **f** The scatter diagram of gene significance (GS) vs module membership (MM) in the brown module of peri-implantitis. Gene GSK3B has been highlighted in red dot, the cutoff of brown module membership = 0.88, the cutoff of gene significance for peri-implantitis = 0.35

We combined GSE33774 and GSE57631 and normalized them. Finally, we obtained the gene expression profiles of 10 normal tissues and 13 peri-implantitis. The WGCNA analysis was designed to search for co-expression gene modules and to explore the association between the gene network and the phenotypes of concern. This time, we downloaded the gene expression information and clinical information of 10 normal tissues and 13 peri-implantitis (Fig. 5a). In this study, we chose the power of β = 32 to construct a scale-free network (Fig. 5b). Finally, after removing the gray module, we obtained 7 gene co-expression modules by combining dynamic tree cutting (Fig. 5c). After combining the module with clinical traits, we found that the brown module was closely related to the disease states (*P* = 0.003), and it also contained the prognostic gene GSK3B (Fig. 5d). In addition, the cluster tree and eigengene adjacency heatmap between modules were shown in Fig. 5e. Finally, we drew the scatter diagram of GS vs MM in the brown module with the peri-implantitis (correlation = 0.44, *P* = 1.4e-27) (Fig. 5f).

### Immune cells in peri-implantitis

**Fig. 6:**
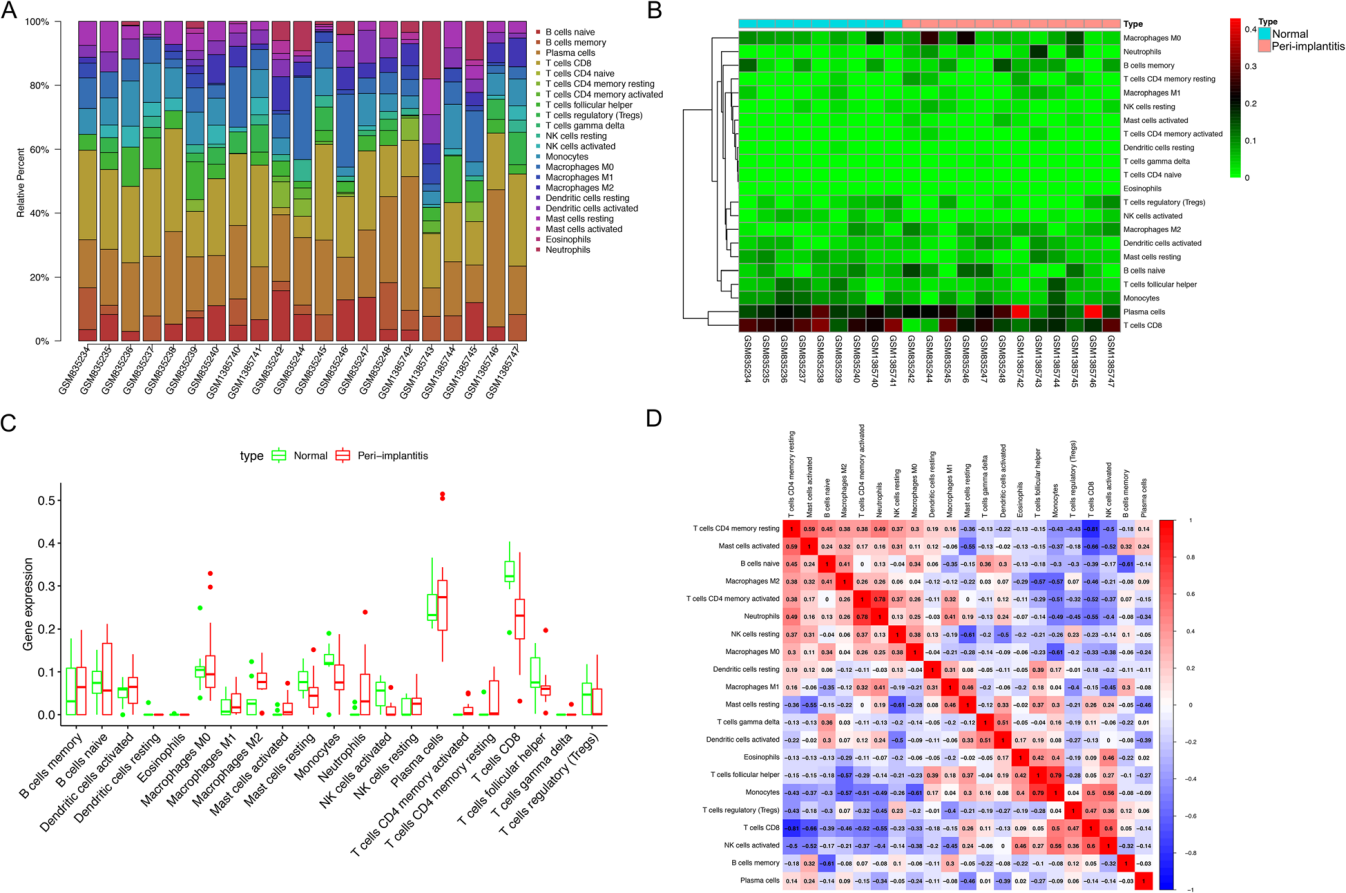
Immune cell composition in peri-implantitis and normal tissues. **a** Composition of infiltrating immune cells in peri-implantitis and normal tissues summarized from calculated mean values for each group. **b** The heatmap shows the differentially infiltrating immune cells between peri-implantitis and normal tissues. **c** The fraction of infiltrating immune cells in peri-implantitis and normal tissues. **d** The correlation heatmap between each immune cell

We predicted the abundance of immune cells in each sample through CIBERSORT, and plotted the corresponding histogram, heatmap and box map of the content of immune cells (Fig. 6a-c). From the figures, we could see the obvious difference between the normal tissue and peri-implantitis. In addition, we also drew the heatmap of the relationship between the corresponding 22 immune cells (Fig. 6d).

### Correlation analysis

**Fig. 7:**
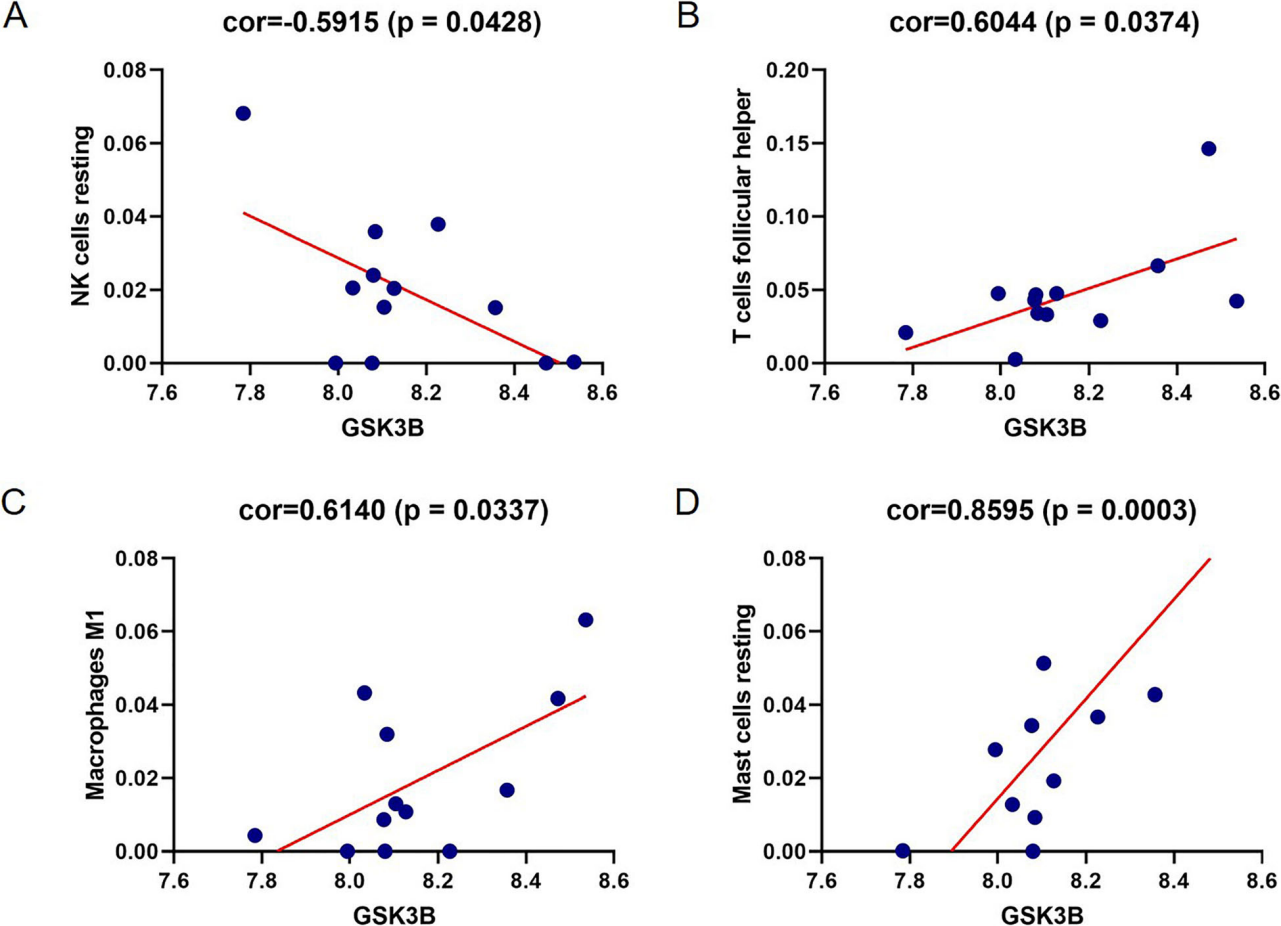
Relationships between the gene expression of GSK3B and infiltration abundance of four types of immune cells. **a** NK cells resting; **b** T cells follicular helper; **c** Macrophages M1; **d** Mast cells resting

We calculated the Pearson correlation to verify the relationship between the abundance of these 22 immune cells and the gene expression of GSK3B. The results showed that GSK3B was associated with four types of immune cells: NK cells resting (correlation = − 0.5915, *P* = 0.0428), T cells follicular helper (correlation = 0.6044, *P* = 0.0374), Macrophages M1 (correlation = 0.614, *P* = 0.0337) and mast cells resting (correlation = 0.8595, *P* = 0.0003) (Fig. 7a-d).

## Discussion

The application of implant denture is a breakthrough in the field of stomatology in recent years. However, peri-implantitis can affect the long-term success rate of dental implants, which is one of the main reasons for the failure of dental implants. Peri-implantitis is a chronic progressive marginal inflammation, which belongs to peri-implant tissue disease. Peri-implant disease includes peri-implant mucositis and peri-implantitis. The former is a reversible disease involving only soft tissue. The latter is the result of further development of mucositis, not only involving soft tissue, but also violated the deep alveolar bone, resulting in bone resorption. If peri-implantitis is not treated in time, it will lead to continuous bone resorption and implant-bone interface separation, which will eventually lead to implant loosening and falling off [18]. The risk factors include periodontitis, smoking, diabetes, poor oral hygiene, systemic disease, keratinized gingival width, IL-1 gene polymorphism, force and implant site. Most scholars believe that bacterial infection is closely related to peri-implantitis [19]. Currently, the treatment of peri-implantitis can be divided into surgical and non-surgical treatment, in which surgical treatment can remove subgingival plaque and sediment, and Guided Bone Regeneration (GBR) surgery can effectively solve the bone resorption problem. However, due to the large surgical trauma, it is easy to cause gingival retraction, and some scholars proposed that only in cases with severe bone resorption and periodontal exploration depth of more than 5 mm after conventional treatment [20]. The treatment of the peri-implantitis is a problem in the current Implantology, and traditional debridement, scaling, anti-bacterial treatment effect is not very satisfactory.

Similar to periodontitis, immune microenvironment plays an important role in the pathogenesis of peri-implantitis, so it is of great significance to understand the immune response and immune microenvironment to clarify the pathogenesis of peri-implantitis and its clinical treatment. Berglundh et al. found that when the detection rate of neutrophils and macrophages in the peri-implant tissues was relatively high in peri-implantitis. In the state of inflammation, pathogenic bacteria invading the tissue would promote the expression of the adhesion molecule ICAM-1 in the peri-implant mucosa and the binding epithelium, forming a certain chemotaxis gradient in the surrounding tissue, which would induce a large number of neutrophils to enter the gingival groove and engulf the invading pathogenic bacteria [21]. Liskmann et al. believed that the secretion of IL-6 by activated Th1 cells played a promoting role, while the secretion of IL-10 by Th2 cells had an anti-inflammatory effect, and the imbalance of the two roles would lead to intensified inflammatory response in the surrounding tissues of implants and the destruction and absorption of alveolar bone [22]. Lin et al. found that activated CD4+ T cells could express the receptor activator of nuclear factor-κB ligand (RANKL), bind to the receptor activator of nuclear factor κB (RANK) on the surface of the osteoclast precursor cells, and promote the maturation, differentiation and bone absorption of osteoclasts [23]. Periodontal pathogens are the initiating factors of peri-implantitis, and the tissue damage caused by the immune response is often more serious than that caused by pathogens themselves. This suggests that in the treatment of peri-implantitis, one should focus on antibacterial, the other should focus on the regulation of immune microenvironment of peri-implantitis, so as to improve the treatment effect to some extent. Therefore, it is expected to provide a new approach for the treatment of peri-implantitis by exploring the role of immune microenvironment in the development of peri-implantitis and proposing more targeted diagnosis and immunotherapy.

CeRNA regulatory network is a newly discovered mechanism for RNA interaction and regulation of coding genes, which extends the previous understanding of the large number of non-coding RNAs in vivo [24]. MiRNAs are newly discovered small molecules that play an important role in gene expression regulation networks. Although miRNAs account for only a small part of the human genome, they are the key regulators of body development and cell homeostasis. MiRNAs are widely expressed in higher eukaryotes and belong to a class of non-coding small RNA molecules. In mammals, miRNAs generally regulate gene expression at the translation level, and the number of miRNAs correlates with the level of translational silencing at each site [25].

This study screened the differentially expressed lncRNAs and IRGs between peri-implantitis and normal tissues and we constructed the corresponding ceRNA network to identify the key hub genes in peri-implantitis. In addition, we conducted GO and KEGG enrichment analysis of IRGs in ceRNA network, and we found that these differentially expressed IRGs were mainly associated with inflammatory processes. Then we verified the DElncRNAs, DEmiRNAs and DEmRNAs obtained in another dataset and found that only GSK3B and miR-1297 existed in both ceRNA networks. Next, we further verified the accuracy of the results. We combined GSE33774 and GSE57631 and conducted WGCNA analysis, which showed that GSK3B appeared in key modules and had an important relationship with peri-implantitis. In order to better detect the immune microenvironment of peri-implantitis, the CIBERSORT was used to evaluate the content of immune cells in normal tissues and peri-implantitis, and Pearson correlation was calculated between the gene expression level of GSK3B and 22 immune cells in peri-implantitis. The results showed that GSK3B was closely related to four types of immune cells.

GSK3B is a serine/threonine kinase originally identified as a regulator of glycogen deposition, the role of which in osteoblasts has been well demonstrated as a negative regulator of β-catenin. It has been reported to play a role in regulating a variety of cellular responses, including cell growth, migration, tumorigenesis and cytokine production [26]. Amirhosseini et al. reported that inhibition of GSK3B could regulate osteoblast and osteoclast differentiation to suppress instable-induced osteolysis and bone loss by activating Wnt/β-catenin signaling [27]. In addition, Jang et al. demonstrated a novel role for GSK3B in regulating RANKL signaling, which was vital for osteoclast differentiation [28]. In this study, it was found that GSK3B was associated with four types of immune cells, especially with the highest correlation with resting mast cells (*P* = 0.0003). Madeleine Radinger et al. determined that GSK3B was constitutively activated in resting human mast cells, which regulated by the phosphorylation status of activating tyrosine residue Y216. In resting human mast cells, they observed constitutive phosphorylation of Y216. However, when the cells were activated, the phosphorylation of this residue have no consistent increase. They found that GSK3B may have functions that precisely control the signal transduction processes required for mast cell chemotaxis and cytokine production [29]. In addition, they also found a small molecule drug that inhibits GSK3B activity could reduce the intensity of allergic inflammatory responses by reducing mast cell survival. They suggested that GSK3B can be a key regulator of mast cell homeostasis through prevention of apoptosis [30]. With regard to miR-1297, a growing number of studies have demonstrated that it may be associated with the pathogenesis and prognosis of a variety of cancers. Bu et al. found that miR-1297 may act as an oncogene by regulating the PTEN/Akt/Skp2 signaling pathway in non-small cell lung cancer (NSCLC) cells [31]. Besides, Chen et al. confirmed that in human cervical cancer tissues, after the overexpression of miR-1297, HeLa cells had an increase in cell proliferation and decrease in apoptosis. PTEN expression was negatively correlated with miR-1297 expression. PTEN silencing showed a similar pattern to the overexpression of miR-1297, which inhibited the growth and apoptosis of HeLa cells in vitro [32]. Through these two ceRNA networks, we found that GSK3B and miR-1297 are interconnected signal axis, and Gao et al. found that GSK3B was the downstream target gene of miR-1297 and growth arrest-specific 5 (GAS5) could be used as a competing endogenous RNA for miR-1297 to weaken its inhibitory effect on GSK3B [33].

This study has some limitations. First of all, this research mainly focuses on data mining and data analysis. These results have not been verified by experiments, and we will further verify the results of this study in the future. In addition, we only obtained a limited datasets and did not include more clinical status of each individual. More datasets will get a reliable result.

## Conclusions

Through this study, we constructed and discovered the immune related ceRNA network and immune infiltration patterns associated with peri-implantitis, and further explored the key genes that play a role in it. We found that GSK3B and miR-1297 may have important significance in the immune microenvironment and pathogenesis of peri-implantitis, which needs to be further proved by later experiments. Our study has built up an immunogenomic landscape with clinical significance for peri-implantitis.

## Supplementary information

Additional file 1: **Table S1.** 14 DElncRNAs interact with 203 DEmiRNAs retrieved from the miRcode database. (DOC 672 kb)

Additional file 2: **Figure S1.** Differentially expressed immune-related genes (IRGs) between peri-implantitis and normal tissues. (TIFF 2575 kb)

## Data Availability

The datasets generated and analyzed during the current study are available in NCBI GEO (https://www.ncbi.nlm.nih.gov/geo/query/acc.cgi?acc=GSE33774 and https://www.ncbi.nlm.nih.gov/geo/query/acc.cgi?acc=GSE57631). The IRG datasets are available in ImmPort database (https://www.immport.org/shared/genelists). The lncRNA-miRNA interactions are available in miRcode database (http://www.mircode.org/). The miRNA-mRNA interactions are available in miRDB (http://mirdb.org/), TargetScan (http://www.targetscan.org/vert_72/) and miRTarBase (http://mirtarbase.mbc.nctu.edu.tw/php/index.php). The data of the DElncRNAs and differentially expressed IRGs in Fig. 1 and Figure S1 are available in GSE33774 (https://www.ncbi.nlm.nih.gov/geo/query/acc.cgi?acc=GSE33774).

## Abbreviations

CeRNA: Competing endogenous RNAs
MiRNAs: MicroRNAs
LncRNAs: Long noncoding RNAs
MRNAs: Message RNAs
MREs: MiRNA response elements
CircRNAs: Circular RNAs
IRGs: Immune-related genes
GEO: Gene Expression Omnibus
DElncRNAs: Differentially expressed lncRNAs
DEGs: Differentially expressed genes
GO: Gene Ontology
KEGG: Kyoto Encyclopedia of Genes and Genomes
FDR: False discovery rate
WGCNA: Weighted gene co-expression network analysis
TOM: Topological overlap matrix
GS: Gene significance
MM: Module membership
DEmRNAs: Differentially expressed mRNAs
BP: Biological process
CC: Cell composition
MF: Molecular function
GBR: Guided Bone Regeneration
RANKL: Receptor activator of nuclear factor-κB ligand
RANK: Receptor activator of nuclear factor κB
GSK3B: Glycogen synthase kinase 3B
NSCLC: Non-small cell lung cancer
GAS5: Growth arrest-specific 5
