# Supplementary material for: Development of an immunogenomic landscape for the competing endogenous RNAs network of peri-implantitis
Source: BMC Med Genet. 2020 Oct 20;21:208. doi: 10.1186/s12881-020-01145-4 (PMC7576812; doi:10.1186/s12881-020-01145-4)
Supplement: Supplementary file 1 — Table S1. 14 DElncRNAs interact with 203 DEmiRNAs retrieved from the miRcode database. (DOC 672 kb) [file 12881_2020_1145_MOESM1_ESM.doc]

**Table S1. 14 DElncRNAs interact with 203 DEmiRNAs retrieved from the miRcode database.**

| lncRNA | miRNA |
| --- | --- |
| FAM182A | hsa-miR-503 |
| FAM182A | hsa-miR-551a |
| FAM182A | hsa-miR-7 |
| FAM182A | hsa-miR-7ab |
| FAM182A | hsa-miR-93 |
| FAM182A | hsa-miR-93a |
| FAM182A | hsa-miR-105 |
| FAM182A | hsa-miR-106a |
| FAM182A | hsa-miR-291a-3p |
| FAM182A | hsa-miR-294 |
| FAM182A | hsa-miR-295 |
| FAM182A | hsa-miR-302abcde |
| FAM182A | hsa-miR-372 |
| FAM182A | hsa-miR-373 |
| FAM182A | hsa-miR-428 |
| FAM182A | hsa-miR-519a |
| FAM182A | hsa-miR-520be |
| FAM182A | hsa-miR-520acd-3p |
| FAM182A | hsa-miR-1378 |
| FAM182A | hsa-miR-1420ac |
| FAM182A | hsa-miR-135ab |
| FAM182A | hsa-miR-135a-5p |
| FAM182A | hsa-miR-139-5p |
| FAM182A | hsa-miR-140 |
| FAM182A | hsa-miR-140-5p |
| FAM182A | hsa-miR-876-3p |
| FAM182A | hsa-miR-1244 |
| FAM182A | hsa-miR-141 |
| FAM182A | hsa-miR-200a |
| FAM182A | hsa-miR-144 |
| FAM182A | hsa-miR-146ac |
| FAM182A | hsa-miR-146b-5p |
| FAM182A | hsa-miR-150 |
| FAM182A | hsa-miR-5127 |
| FAM182A | hsa-miR-153 |
| FAM182A | hsa-miR-155 |
| FAM182A | hsa-miR-17 |
| FAM182A | hsa-miR-17-5p |
| FAM182A | hsa-miR-20ab |
| FAM182A | hsa-miR-20b-5p |
| FAM182A | hsa-miR-106ab |
| FAM182A | hsa-miR-427 |
| FAM182A | hsa-miR-518a-3p |
| FAM182A | hsa-miR-519d |
| FAM182A | hsa-miR-181abcd |
| FAM182A | hsa-miR-4262 |
| FAM182A | hsa-miR-18ab |
| FAM182A | hsa-miR-4735-3p |
| FAM182A | hsa-miR-196abc |
| FAM182A | hsa-miR-199ab-5p |
| FAM182A | hsa-miR-203 |
| FAM182A | hsa-miR-205 |
| FAM182A | hsa-miR-205ab |
| FAM182A | hsa-miR-214 |
| FAM182A | hsa-miR-761 |
| FAM182A | hsa-miR-3619-5p |
| FAM182A | hsa-miR-223 |
| FAM182A | hsa-miR-23abc |
| FAM182A | hsa-miR-23b-3p |
| FAM182A | hsa-miR-24 |
| FAM182A | hsa-miR-24ab |
| FAM182A | hsa-miR-24-3p |
| FAM182A | hsa-miR-25 |
| FAM182A | hsa-miR-32 |
| FAM182A | hsa-miR-92abc |
| FAM182A | hsa-miR-363 |
| FAM182A | hsa-miR-363-3p |
| FAM182A | hsa-miR-367 |
| FAM182A | hsa-miR-26ab |
| FAM182A | hsa-miR-1297 |
| FAM182A | hsa-miR-4465 |
| FAM182A | hsa-miR-27abc |
| FAM182A | hsa-miR-27a-3p |
| FAM182A | hsa-miR-29abcd |
| FAM182A | hsa-miR-30abcdef |
| FAM182A | hsa-miR-30abe-5p |
| FAM182A | hsa-miR-384-5p |
| FAM182A | hsa-miR-103a |
| FAM182A | hsa-miR-107 |
| FAM182A | hsa-miR-107ab |
| FAM182A | hsa-miR-124 |
| FAM182A | hsa-miR-124ab |
| FAM182A | hsa-miR-506 |
| FAM182A | hsa-miR-338 |
| FAM182A | hsa-miR-338-3p |
| FAM182A | hsa-miR-34ac |
| FAM182A | hsa-miR-34bc-5p |
| FAM182A | hsa-miR-449abc |
| FAM182A | hsa-miR-449c-5p |
| FAM182A | hsa-miR-425 |
| FAM182A | hsa-miR-425-5p |
| FAM182A | hsa-miR-489 |
| FAM182A | hsa-miR-10abc |
| FAM182A | hsa-miR-10a-5p |
| FAM182A | hsa-miR-128 |
| FAM182A | hsa-miR-128ab |
| FAM182A | hsa-miR-490-3p |
| C7orf69 | hsa-miR-223 |
| C7orf69 | hsa-miR-26ab |
| C7orf69 | hsa-miR-1297 |
| C7orf69 | hsa-miR-4465 |
| C7orf69 | hsa-miR-31 |
| C7orf69 | hsa-miR-124 |
| C7orf69 | hsa-miR-124ab |
| C7orf69 | hsa-miR-506 |
| C7orf69 | hsa-miR-383 |
| SMAD5-AS1 | hsa-miR-503 |
| SMAD5-AS1 | hsa-miR-9 |
| SMAD5-AS1 | hsa-miR-9ab |
| SMAD5-AS1 | hsa-miR-135ab |
| SMAD5-AS1 | hsa-miR-135a-5p |
| SMAD5-AS1 | hsa-miR-139-5p |
| SMAD5-AS1 | hsa-miR-145 |
| SMAD5-AS1 | hsa-miR-150 |
| SMAD5-AS1 | hsa-miR-5127 |
| SMAD5-AS1 | hsa-miR-15abc |
| SMAD5-AS1 | hsa-miR-16 |
| SMAD5-AS1 | hsa-miR-16abc |
| SMAD5-AS1 | hsa-miR-195 |
| SMAD5-AS1 | hsa-miR-322 |
| SMAD5-AS1 | hsa-miR-424 |
| SMAD5-AS1 | hsa-miR-497 |
| SMAD5-AS1 | hsa-miR-1907 |
| SMAD5-AS1 | hsa-miR-181abcd |
| SMAD5-AS1 | hsa-miR-4262 |
| SMAD5-AS1 | hsa-miR-204 |
| SMAD5-AS1 | hsa-miR-204b |
| SMAD5-AS1 | hsa-miR-211 |
| SMAD5-AS1 | hsa-miR-205 |
| SMAD5-AS1 | hsa-miR-205ab |
| SMAD5-AS1 | hsa-miR-21 |
| SMAD5-AS1 | hsa-miR-590-5p |
| SMAD5-AS1 | hsa-miR-217 |
| SMAD5-AS1 | hsa-miR-218 |
| SMAD5-AS1 | hsa-miR-218a |
| SMAD5-AS1 | hsa-miR-219-5p |
| SMAD5-AS1 | hsa-miR-508 |
| SMAD5-AS1 | hsa-miR-508-3p |
| SMAD5-AS1 | hsa-miR-4782-3p |
| SMAD5-AS1 | hsa-miR-223 |
| SMAD5-AS1 | hsa-miR-23abc |
| SMAD5-AS1 | hsa-miR-23b-3p |
| SMAD5-AS1 | hsa-miR-101 |
| SMAD5-AS1 | hsa-miR-101ab |
| SMAD5-AS1 | hsa-miR-30abcdef |
| SMAD5-AS1 | hsa-miR-30abe-5p |
| SMAD5-AS1 | hsa-miR-384-5p |
| SMAD5-AS1 | hsa-miR-125a-5p |
| SMAD5-AS1 | hsa-miR-125b-5p |
| SMAD5-AS1 | hsa-miR-351 |
| SMAD5-AS1 | hsa-miR-670 |
| SMAD5-AS1 | hsa-miR-4319 |
| SMAD5-AS1 | hsa-miR-455-5p |
| SMAD5-AS1 | hsa-miR-129-5p |
| SMAD5-AS1 | hsa-miR-129ab-5p |
| SHANK2-AS3 | hsa-miR-96 |
| SHANK2-AS3 | hsa-miR-507 |
| SHANK2-AS3 | hsa-miR-1271 |
| SHANK2-AS3 | hsa-miR-145 |
| SHANK2-AS3 | hsa-miR-146ac |
| SHANK2-AS3 | hsa-miR-146b-5p |
| SHANK2-AS3 | hsa-miR-150 |
| SHANK2-AS3 | hsa-miR-5127 |
| SHANK2-AS3 | hsa-miR-187 |
| SHANK2-AS3 | hsa-miR-18ab |
| SHANK2-AS3 | hsa-miR-4735-3p |
| SHANK2-AS3 | hsa-miR-19ab |
| SHANK2-AS3 | hsa-miR-204 |
| SHANK2-AS3 | hsa-miR-204b |
| SHANK2-AS3 | hsa-miR-211 |
| SHANK2-AS3 | hsa-miR-205 |
| SHANK2-AS3 | hsa-miR-205ab |
| SHANK2-AS3 | hsa-miR-208ab |
| SHANK2-AS3 | hsa-miR-208ab-3p |
| SHANK2-AS3 | hsa-miR-218 |
| SHANK2-AS3 | hsa-miR-218a |
| SHANK2-AS3 | hsa-miR-122 |
| SHANK2-AS3 | hsa-miR-122a |
| SHANK2-AS3 | hsa-miR-1352 |
| SHANK2-AS3 | hsa-miR-24 |
| SHANK2-AS3 | hsa-miR-24ab |
| SHANK2-AS3 | hsa-miR-24-3p |
| SHANK2-AS3 | hsa-miR-27abc |
| SHANK2-AS3 | hsa-miR-27a-3p |
| SHANK2-AS3 | hsa-miR-499-5p |
| MIR31HG | hsa-miR-7 |
| MIR31HG | hsa-miR-7ab |
| MIR31HG | hsa-miR-193 |
| MIR31HG | hsa-miR-193b |
| MIR31HG | hsa-miR-193a-3p |
| MIR31HG | hsa-miR-1ab |
| MIR31HG | hsa-miR-206 |
| MIR31HG | hsa-miR-613 |
| MIR31HG | hsa-miR-214 |
| MIR31HG | hsa-miR-761 |
| MIR31HG | hsa-miR-3619-5p |
| MIR31HG | hsa-miR-27abc |
| MIR31HG | hsa-miR-27a-3p |
| MIR31HG | hsa-miR-34ac |
| MIR31HG | hsa-miR-34bc-5p |
| MIR31HG | hsa-miR-449abc |
| MIR31HG | hsa-miR-449c-5p |
| FAM182B | hsa-miR-551a |
| FAM182B | hsa-miR-143 |
| FAM182B | hsa-miR-1721 |
| FAM182B | hsa-miR-4770 |
| FAM182B | hsa-miR-144 |
| FAM182B | hsa-miR-146ac |
| FAM182B | hsa-miR-146b-5p |
| FAM182B | hsa-miR-193 |
| FAM182B | hsa-miR-193b |
| FAM182B | hsa-miR-193a-3p |
| FAM182B | hsa-miR-199ab-5p |
| FAM182B | hsa-miR-1ab |
| FAM182B | hsa-miR-206 |
| FAM182B | hsa-miR-613 |
| FAM182B | hsa-miR-203 |
| FAM182B | hsa-miR-218 |
| FAM182B | hsa-miR-218a |
| FAM182B | hsa-miR-25 |
| FAM182B | hsa-miR-32 |
| FAM182B | hsa-miR-92abc |
| FAM182B | hsa-miR-363 |
| FAM182B | hsa-miR-363-3p |
| FAM182B | hsa-miR-367 |
| FAM182B | hsa-miR-29abcd |
| FAM182B | hsa-miR-103a |
| FAM182B | hsa-miR-107 |
| FAM182B | hsa-miR-107ab |
| FAM182B | hsa-miR-34ac |
| FAM182B | hsa-miR-34bc-5p |
| FAM182B | hsa-miR-449abc |
| FAM182B | hsa-miR-449c-5p |
| FAM182B | hsa-miR-125a-5p |
| FAM182B | hsa-miR-125b-5p |
| FAM182B | hsa-miR-351 |
| FAM182B | hsa-miR-670 |
| FAM182B | hsa-miR-4319 |
| FAM182B | hsa-miR-129-5p |
| FAM182B | hsa-miR-129ab-5p |
| TTTY14 | hsa-miR-133abc |
| TTTY14 | hsa-miR-93 |
| TTTY14 | hsa-miR-93a |
| TTTY14 | hsa-miR-105 |
| TTTY14 | hsa-miR-106a |
| TTTY14 | hsa-miR-291a-3p |
| TTTY14 | hsa-miR-294 |
| TTTY14 | hsa-miR-295 |
| TTTY14 | hsa-miR-302abcde |
| TTTY14 | hsa-miR-372 |
| TTTY14 | hsa-miR-373 |
| TTTY14 | hsa-miR-428 |
| TTTY14 | hsa-miR-519a |
| TTTY14 | hsa-miR-520be |
| TTTY14 | hsa-miR-520acd-3p |
| TTTY14 | hsa-miR-1378 |
| TTTY14 | hsa-miR-1420ac |
| TTTY14 | hsa-miR-137 |
| TTTY14 | hsa-miR-137ab |
| TTTY14 | hsa-miR-138 |
| TTTY14 | hsa-miR-138ab |
| TTTY14 | hsa-miR-146ac |
| TTTY14 | hsa-miR-146b-5p |
| TTTY14 | hsa-miR-150 |
| TTTY14 | hsa-miR-5127 |
| TTTY14 | hsa-miR-17 |
| TTTY14 | hsa-miR-17-5p |
| TTTY14 | hsa-miR-20ab |
| TTTY14 | hsa-miR-20b-5p |
| TTTY14 | hsa-miR-106ab |
| TTTY14 | hsa-miR-427 |
| TTTY14 | hsa-miR-518a-3p |
| TTTY14 | hsa-miR-519d |
| TTTY14 | hsa-miR-184 |
| TTTY14 | hsa-miR-203 |
| TTTY14 | hsa-miR-204 |
| TTTY14 | hsa-miR-204b |
| TTTY14 | hsa-miR-211 |
| TTTY14 | hsa-miR-205 |
| TTTY14 | hsa-miR-205ab |
| TTTY14 | hsa-miR-217 |
| TTTY14 | hsa-miR-219-5p |
| TTTY14 | hsa-miR-508 |
| TTTY14 | hsa-miR-508-3p |
| TTTY14 | hsa-miR-4782-3p |
| TTTY14 | hsa-miR-22 |
| TTTY14 | hsa-miR-22-3p |
| TTTY14 | hsa-miR-24 |
| TTTY14 | hsa-miR-24ab |
| TTTY14 | hsa-miR-24-3p |
| TTTY14 | hsa-miR-26ab |
| TTTY14 | hsa-miR-1297 |
| TTTY14 | hsa-miR-4465 |
| TTTY14 | hsa-miR-30abcdef |
| TTTY14 | hsa-miR-30abe-5p |
| TTTY14 | hsa-miR-384-5p |
| TTTY14 | hsa-miR-31 |
| TTTY14 | hsa-miR-338 |
| TTTY14 | hsa-miR-338-3p |
| TTTY14 | hsa-miR-33ab |
| TTTY14 | hsa-miR-33-5p |
| TTTY14 | hsa-miR-125a-5p |
| TTTY14 | hsa-miR-125b-5p |
| TTTY14 | hsa-miR-351 |
| TTTY14 | hsa-miR-670 |
| TTTY14 | hsa-miR-4319 |
| TTTY14 | hsa-miR-10abc |
| TTTY14 | hsa-miR-10a-5p |
| TTTY14 | hsa-miR-129-5p |
| TTTY14 | hsa-miR-129ab-5p |
| TTTY14 | hsa-miR-490-3p |
| TTTY14 | hsa-miR-499-5p |
| MRVI1-AS1 | hsa-miR-137 |
| MRVI1-AS1 | hsa-miR-137ab |
| MRVI1-AS1 | hsa-miR-148ab-3p |
| MRVI1-AS1 | hsa-miR-152 |
| MRVI1-AS1 | hsa-miR-183 |
| MRVI1-AS1 | hsa-miR-let-7 |
| MRVI1-AS1 | hsa-miR-98 |
| MRVI1-AS1 | hsa-miR-4458 |
| MRVI1-AS1 | hsa-miR-4500 |
| MRVI1-AS1 | hsa-miR-19ab |
| MRVI1-AS1 | hsa-miR-205 |
| MRVI1-AS1 | hsa-miR-205ab |
| MRVI1-AS1 | hsa-miR-214 |
| MRVI1-AS1 | hsa-miR-761 |
| MRVI1-AS1 | hsa-miR-3619-5p |
| MRVI1-AS1 | hsa-miR-218 |
| MRVI1-AS1 | hsa-miR-218a |
| MRVI1-AS1 | hsa-miR-221 |
| MRVI1-AS1 | hsa-miR-222 |
| MRVI1-AS1 | hsa-miR-222ab |
| MRVI1-AS1 | hsa-miR-1928 |
| MRVI1-AS1 | hsa-miR-27abc |
| MRVI1-AS1 | hsa-miR-27a-3p |
| MRVI1-AS1 | hsa-miR-103a |
| MRVI1-AS1 | hsa-miR-107 |
| MRVI1-AS1 | hsa-miR-107ab |
| MRVI1-AS1 | hsa-miR-338 |
| MRVI1-AS1 | hsa-miR-338-3p |
| MRVI1-AS1 | hsa-miR-10abc |
| MRVI1-AS1 | hsa-miR-10a-5p |
| CASC2 | hsa-miR-130ac |
| CASC2 | hsa-miR-301ab |
| CASC2 | hsa-miR-301b |
| CASC2 | hsa-miR-301b-3p |
| CASC2 | hsa-miR-454 |
| CASC2 | hsa-miR-721 |
| CASC2 | hsa-miR-4295 |
| CASC2 | hsa-miR-3666 |
| CASC2 | hsa-miR-132 |
| CASC2 | hsa-miR-212 |
| CASC2 | hsa-miR-212-3p |
| CASC2 | hsa-miR-133abc |
| CASC2 | hsa-miR-9 |
| CASC2 | hsa-miR-9ab |
| CASC2 | hsa-miR-93 |
| CASC2 | hsa-miR-93a |
| CASC2 | hsa-miR-105 |
| CASC2 | hsa-miR-106a |
| CASC2 | hsa-miR-291a-3p |
| CASC2 | hsa-miR-294 |
| CASC2 | hsa-miR-295 |
| CASC2 | hsa-miR-302abcde |
| CASC2 | hsa-miR-372 |
| CASC2 | hsa-miR-373 |
| CASC2 | hsa-miR-428 |
| CASC2 | hsa-miR-519a |
| CASC2 | hsa-miR-520be |
| CASC2 | hsa-miR-520acd-3p |
| CASC2 | hsa-miR-1378 |
| CASC2 | hsa-miR-1420ac |
| CASC2 | hsa-miR-135ab |
| CASC2 | hsa-miR-135a-5p |
| CASC2 | hsa-miR-138 |
| CASC2 | hsa-miR-138ab |
| CASC2 | hsa-miR-140 |
| CASC2 | hsa-miR-140-5p |
| CASC2 | hsa-miR-876-3p |
| CASC2 | hsa-miR-1244 |
| CASC2 | hsa-miR-141 |
| CASC2 | hsa-miR-200a |
| CASC2 | hsa-miR-143 |
| CASC2 | hsa-miR-1721 |
| CASC2 | hsa-miR-4770 |
| CASC2 | hsa-miR-144 |
| CASC2 | hsa-miR-148ab-3p |
| CASC2 | hsa-miR-152 |
| CASC2 | hsa-miR-150 |
| CASC2 | hsa-miR-5127 |
| CASC2 | hsa-miR-153 |
| CASC2 | hsa-miR-155 |
| CASC2 | hsa-miR-15abc |
| CASC2 | hsa-miR-16 |
| CASC2 | hsa-miR-16abc |
| CASC2 | hsa-miR-195 |
| CASC2 | hsa-miR-322 |
| CASC2 | hsa-miR-424 |
| CASC2 | hsa-miR-497 |
| CASC2 | hsa-miR-1907 |
| CASC2 | hsa-miR-17 |
| CASC2 | hsa-miR-17-5p |
| CASC2 | hsa-miR-20ab |
| CASC2 | hsa-miR-20b-5p |
| CASC2 | hsa-miR-106ab |
| CASC2 | hsa-miR-427 |
| CASC2 | hsa-miR-518a-3p |
| CASC2 | hsa-miR-519d |
| CASC2 | hsa-miR-181abcd |
| CASC2 | hsa-miR-4262 |
| CASC2 | hsa-miR-183 |
| CASC2 | hsa-miR-let-7 |
| CASC2 | hsa-miR-98 |
| CASC2 | hsa-miR-4458 |
| CASC2 | hsa-miR-4500 |
| CASC2 | hsa-miR-18ab |
| CASC2 | hsa-miR-4735-3p |
| CASC2 | hsa-miR-192 |
| CASC2 | hsa-miR-215 |
| CASC2 | hsa-miR-193 |
| CASC2 | hsa-miR-193b |
| CASC2 | hsa-miR-193a-3p |
| CASC2 | hsa-miR-194 |
| CASC2 | hsa-miR-199ab-5p |
| CASC2 | hsa-miR-19ab |
| CASC2 | hsa-miR-1ab |
| CASC2 | hsa-miR-206 |
| CASC2 | hsa-miR-613 |
| CASC2 | hsa-miR-200bc |
| CASC2 | hsa-miR-429 |
| CASC2 | hsa-miR-548a |
| CASC2 | hsa-miR-203 |
| CASC2 | hsa-miR-204 |
| CASC2 | hsa-miR-204b |
| CASC2 | hsa-miR-211 |
| CASC2 | hsa-miR-205 |
| CASC2 | hsa-miR-205ab |
| CASC2 | hsa-miR-208ab |
| CASC2 | hsa-miR-208ab-3p |
| CASC2 | hsa-miR-21 |
| CASC2 | hsa-miR-590-5p |
| CASC2 | hsa-miR-214 |
| CASC2 | hsa-miR-761 |
| CASC2 | hsa-miR-3619-5p |
| CASC2 | hsa-miR-216a |
| CASC2 | hsa-miR-216b |
| CASC2 | hsa-miR-216b-5p |
| CASC2 | hsa-miR-217 |
| CASC2 | hsa-miR-218 |
| CASC2 | hsa-miR-218a |
| CASC2 | hsa-miR-221 |
| CASC2 | hsa-miR-222 |
| CASC2 | hsa-miR-222ab |
| CASC2 | hsa-miR-1928 |
| CASC2 | hsa-miR-23abc |
| CASC2 | hsa-miR-23b-3p |
| CASC2 | hsa-miR-24 |
| CASC2 | hsa-miR-24ab |
| CASC2 | hsa-miR-24-3p |
| CASC2 | hsa-miR-25 |
| CASC2 | hsa-miR-32 |
| CASC2 | hsa-miR-92abc |
| CASC2 | hsa-miR-363 |
| CASC2 | hsa-miR-363-3p |
| CASC2 | hsa-miR-367 |
| CASC2 | hsa-miR-27abc |
| CASC2 | hsa-miR-27a-3p |
| CASC2 | hsa-miR-101 |
| CASC2 | hsa-miR-101ab |
| CASC2 | hsa-miR-29abcd |
| CASC2 | hsa-miR-31 |
| CASC2 | hsa-miR-103a |
| CASC2 | hsa-miR-107 |
| CASC2 | hsa-miR-107ab |
| CASC2 | hsa-miR-124 |
| CASC2 | hsa-miR-124ab |
| CASC2 | hsa-miR-506 |
| CASC2 | hsa-miR-338 |
| CASC2 | hsa-miR-338-3p |
| CASC2 | hsa-miR-33ab |
| CASC2 | hsa-miR-33-5p |
| CASC2 | hsa-miR-34ac |
| CASC2 | hsa-miR-34bc-5p |
| CASC2 | hsa-miR-449abc |
| CASC2 | hsa-miR-449c-5p |
| CASC2 | hsa-miR-383 |
| CASC2 | hsa-miR-425 |
| CASC2 | hsa-miR-425-5p |
| CASC2 | hsa-miR-489 |
| CASC2 | hsa-miR-125a-5p |
| CASC2 | hsa-miR-125b-5p |
| CASC2 | hsa-miR-351 |
| CASC2 | hsa-miR-670 |
| CASC2 | hsa-miR-4319 |
| CASC2 | hsa-miR-128 |
| CASC2 | hsa-miR-128ab |
| CASC2 | hsa-miR-490-3p |
| CASC2 | hsa-miR-499-5p |
| KTN1-AS1 | hsa-miR-503 |
| KTN1-AS1 | hsa-miR-7 |
| KTN1-AS1 | hsa-miR-7ab |
| KTN1-AS1 | hsa-miR-9 |
| KTN1-AS1 | hsa-miR-9ab |
| KTN1-AS1 | hsa-miR-93 |
| KTN1-AS1 | hsa-miR-93a |
| KTN1-AS1 | hsa-miR-105 |
| KTN1-AS1 | hsa-miR-106a |
| KTN1-AS1 | hsa-miR-291a-3p |
| KTN1-AS1 | hsa-miR-294 |
| KTN1-AS1 | hsa-miR-295 |
| KTN1-AS1 | hsa-miR-302abcde |
| KTN1-AS1 | hsa-miR-372 |
| KTN1-AS1 | hsa-miR-373 |
| KTN1-AS1 | hsa-miR-428 |
| KTN1-AS1 | hsa-miR-519a |
| KTN1-AS1 | hsa-miR-520be |
| KTN1-AS1 | hsa-miR-520acd-3p |
| KTN1-AS1 | hsa-miR-1378 |
| KTN1-AS1 | hsa-miR-1420ac |
| KTN1-AS1 | hsa-miR-99ab |
| KTN1-AS1 | hsa-miR-100 |
| KTN1-AS1 | hsa-miR-139-5p |
| KTN1-AS1 | hsa-miR-143 |
| KTN1-AS1 | hsa-miR-1721 |
| KTN1-AS1 | hsa-miR-4770 |
| KTN1-AS1 | hsa-miR-145 |
| KTN1-AS1 | hsa-miR-146ac |
| KTN1-AS1 | hsa-miR-146b-5p |
| KTN1-AS1 | hsa-miR-148ab-3p |
| KTN1-AS1 | hsa-miR-152 |
| KTN1-AS1 | hsa-miR-150 |
| KTN1-AS1 | hsa-miR-5127 |
| KTN1-AS1 | hsa-miR-153 |
| KTN1-AS1 | hsa-miR-17 |
| KTN1-AS1 | hsa-miR-17-5p |
| KTN1-AS1 | hsa-miR-20ab |
| KTN1-AS1 | hsa-miR-20b-5p |
| KTN1-AS1 | hsa-miR-106ab |
| KTN1-AS1 | hsa-miR-427 |
| KTN1-AS1 | hsa-miR-518a-3p |
| KTN1-AS1 | hsa-miR-519d |
| KTN1-AS1 | hsa-miR-182 |
| KTN1-AS1 | hsa-miR-18ab |
| KTN1-AS1 | hsa-miR-4735-3p |
| KTN1-AS1 | hsa-miR-199ab-5p |
| KTN1-AS1 | hsa-miR-203 |
| KTN1-AS1 | hsa-miR-204 |
| KTN1-AS1 | hsa-miR-204b |
| KTN1-AS1 | hsa-miR-211 |
| KTN1-AS1 | hsa-miR-214 |
| KTN1-AS1 | hsa-miR-761 |
| KTN1-AS1 | hsa-miR-3619-5p |
| KTN1-AS1 | hsa-miR-216b |
| KTN1-AS1 | hsa-miR-216b-5p |
| KTN1-AS1 | hsa-miR-218 |
| KTN1-AS1 | hsa-miR-218a |
| KTN1-AS1 | hsa-miR-23abc |
| KTN1-AS1 | hsa-miR-23b-3p |
| KTN1-AS1 | hsa-miR-24 |
| KTN1-AS1 | hsa-miR-24ab |
| KTN1-AS1 | hsa-miR-24-3p |
| KTN1-AS1 | hsa-miR-26ab |
| KTN1-AS1 | hsa-miR-1297 |
| KTN1-AS1 | hsa-miR-4465 |
| KTN1-AS1 | hsa-miR-338 |
| KTN1-AS1 | hsa-miR-338-3p |
| KTN1-AS1 | hsa-miR-33ab |
| KTN1-AS1 | hsa-miR-33-5p |
| KTN1-AS1 | hsa-miR-34ac |
| KTN1-AS1 | hsa-miR-34bc-5p |
| KTN1-AS1 | hsa-miR-449abc |
| KTN1-AS1 | hsa-miR-449c-5p |
| KTN1-AS1 | hsa-miR-425 |
| KTN1-AS1 | hsa-miR-425-5p |
| KTN1-AS1 | hsa-miR-489 |
| KTN1-AS1 | hsa-miR-125a-5p |
| KTN1-AS1 | hsa-miR-125b-5p |
| KTN1-AS1 | hsa-miR-351 |
| KTN1-AS1 | hsa-miR-670 |
| KTN1-AS1 | hsa-miR-4319 |
| KTN1-AS1 | hsa-miR-451 |
| KTN1-AS1 | hsa-miR-455-5p |
| KTN1-AS1 | hsa-miR-128 |
| KTN1-AS1 | hsa-miR-128ab |
| KTN1-AS1 | hsa-miR-129-5p |
| KTN1-AS1 | hsa-miR-129ab-5p |
| SNHG12 | hsa-miR-503 |
| SNHG12 | hsa-miR-133abc |
| SNHG12 | hsa-miR-9 |
| SNHG12 | hsa-miR-9ab |
| SNHG12 | hsa-miR-138 |
| SNHG12 | hsa-miR-138ab |
| SNHG12 | hsa-miR-140 |
| SNHG12 | hsa-miR-140-5p |
| SNHG12 | hsa-miR-876-3p |
| SNHG12 | hsa-miR-1244 |
| SNHG12 | hsa-miR-146ac |
| SNHG12 | hsa-miR-146b-5p |
| SNHG12 | hsa-miR-148ab-3p |
| SNHG12 | hsa-miR-152 |
| SNHG12 | hsa-miR-150 |
| SNHG12 | hsa-miR-5127 |
| SNHG12 | hsa-miR-15abc |
| SNHG12 | hsa-miR-16 |
| SNHG12 | hsa-miR-16abc |
| SNHG12 | hsa-miR-195 |
| SNHG12 | hsa-miR-322 |
| SNHG12 | hsa-miR-424 |
| SNHG12 | hsa-miR-497 |
| SNHG12 | hsa-miR-1907 |
| SNHG12 | hsa-miR-181abcd |
| SNHG12 | hsa-miR-4262 |
| SNHG12 | hsa-miR-let-7 |
| SNHG12 | hsa-miR-98 |
| SNHG12 | hsa-miR-4458 |
| SNHG12 | hsa-miR-4500 |
| SNHG12 | hsa-miR-187 |
| SNHG12 | hsa-miR-193 |
| SNHG12 | hsa-miR-193b |
| SNHG12 | hsa-miR-193a-3p |
| SNHG12 | hsa-miR-194 |
| SNHG12 | hsa-miR-199ab-5p |
| SNHG12 | hsa-miR-1ab |
| SNHG12 | hsa-miR-206 |
| SNHG12 | hsa-miR-613 |
| SNHG12 | hsa-miR-200bc |
| SNHG12 | hsa-miR-429 |
| SNHG12 | hsa-miR-548a |
| SNHG12 | hsa-miR-204 |
| SNHG12 | hsa-miR-204b |
| SNHG12 | hsa-miR-211 |
| SNHG12 | hsa-miR-208ab |
| SNHG12 | hsa-miR-208ab-3p |
| SNHG12 | hsa-miR-217 |
| SNHG12 | hsa-miR-218 |
| SNHG12 | hsa-miR-218a |
| SNHG12 | hsa-miR-219-5p |
| SNHG12 | hsa-miR-508 |
| SNHG12 | hsa-miR-508-3p |
| SNHG12 | hsa-miR-4782-3p |
| SNHG12 | hsa-miR-122 |
| SNHG12 | hsa-miR-122a |
| SNHG12 | hsa-miR-1352 |
| SNHG12 | hsa-miR-24 |
| SNHG12 | hsa-miR-24ab |
| SNHG12 | hsa-miR-24-3p |
| SNHG12 | hsa-miR-338 |
| SNHG12 | hsa-miR-338-3p |
| SNHG12 | hsa-miR-33ab |
| SNHG12 | hsa-miR-33-5p |
| SNHG12 | hsa-miR-425 |
| SNHG12 | hsa-miR-425-5p |
| SNHG12 | hsa-miR-489 |
| SNHG12 | hsa-miR-10abc |
| SNHG12 | hsa-miR-10a-5p |
| SNHG12 | hsa-miR-451 |
| SNHG12 | hsa-miR-129-5p |
| SNHG12 | hsa-miR-129ab-5p |
| SNHG12 | hsa-miR-499-5p |
| TTTY10 | hsa-miR-130ac |
| TTTY10 | hsa-miR-301ab |
| TTTY10 | hsa-miR-301b |
| TTTY10 | hsa-miR-301b-3p |
| TTTY10 | hsa-miR-454 |
| TTTY10 | hsa-miR-721 |
| TTTY10 | hsa-miR-4295 |
| TTTY10 | hsa-miR-3666 |
| TTTY10 | hsa-miR-135ab |
| TTTY10 | hsa-miR-135a-5p |
| TTTY10 | hsa-miR-139-5p |
| TTTY10 | hsa-miR-141 |
| TTTY10 | hsa-miR-200a |
| TTTY10 | hsa-miR-143 |
| TTTY10 | hsa-miR-1721 |
| TTTY10 | hsa-miR-4770 |
| TTTY10 | hsa-miR-148ab-3p |
| TTTY10 | hsa-miR-152 |
| TTTY10 | hsa-miR-155 |
| TTTY10 | hsa-miR-17 |
| TTTY10 | hsa-miR-17-5p |
| TTTY10 | hsa-miR-20ab |
| TTTY10 | hsa-miR-20b-5p |
| TTTY10 | hsa-miR-93 |
| TTTY10 | hsa-miR-106ab |
| TTTY10 | hsa-miR-427 |
| TTTY10 | hsa-miR-518a-3p |
| TTTY10 | hsa-miR-519d |
| TTTY10 | hsa-miR-190 |
| TTTY10 | hsa-miR-190ab |
| TTTY10 | hsa-miR-194 |
| TTTY10 | hsa-miR-199ab-5p |
| TTTY10 | hsa-miR-203 |
| TTTY10 | hsa-miR-205 |
| TTTY10 | hsa-miR-205ab |
| TTTY10 | hsa-miR-218 |
| TTTY10 | hsa-miR-218a |
| TTTY10 | hsa-miR-24 |
| TTTY10 | hsa-miR-24ab |
| TTTY10 | hsa-miR-24-3p |
| TTTY10 | hsa-miR-27abc |
| TTTY10 | hsa-miR-27a-3p |
| TTTY10 | hsa-miR-338 |
| TTTY10 | hsa-miR-338-3p |
| TTTY10 | hsa-miR-33a-3p |
| TTTY10 | hsa-miR-365 |
| TTTY10 | hsa-miR-365-3p |
| TTTY10 | hsa-miR-383 |
| TTTY10 | hsa-miR-125a-5p |
| TTTY10 | hsa-miR-125b-5p |
| TTTY10 | hsa-miR-351 |
| TTTY10 | hsa-miR-670 |
| TTTY10 | hsa-miR-4319 |
| TTTY10 | hsa-miR-128 |
| TTTY10 | hsa-miR-128ab |
| TTTY10 | hsa-miR-490-3p |
| DLEU2 | hsa-miR-551a |
| DLEU2 | hsa-miR-7 |
| DLEU2 | hsa-miR-7ab |
| DLEU2 | hsa-miR-133abc |
| DLEU2 | hsa-miR-9 |
| DLEU2 | hsa-miR-9ab |
| DLEU2 | hsa-miR-96 |
| DLEU2 | hsa-miR-507 |
| DLEU2 | hsa-miR-1271 |
| DLEU2 | hsa-miR-137 |
| DLEU2 | hsa-miR-137ab |
| DLEU2 | hsa-miR-141 |
| DLEU2 | hsa-miR-200a |
| DLEU2 | hsa-miR-143 |
| DLEU2 | hsa-miR-1721 |
| DLEU2 | hsa-miR-4770 |
| DLEU2 | hsa-miR-144 |
| DLEU2 | hsa-miR-150 |
| DLEU2 | hsa-miR-5127 |
| DLEU2 | hsa-miR-153 |
| DLEU2 | hsa-miR-181abcd |
| DLEU2 | hsa-miR-4262 |
| DLEU2 | hsa-miR-182 |
| DLEU2 | hsa-miR-let-7 |
| DLEU2 | hsa-miR-98 |
| DLEU2 | hsa-miR-4458 |
| DLEU2 | hsa-miR-4500 |
| DLEU2 | hsa-miR-194 |
| DLEU2 | hsa-miR-1ab |
| DLEU2 | hsa-miR-206 |
| DLEU2 | hsa-miR-613 |
| DLEU2 | hsa-miR-203 |
| DLEU2 | hsa-miR-205 |
| DLEU2 | hsa-miR-205ab |
| DLEU2 | hsa-miR-21 |
| DLEU2 | hsa-miR-590-5p |
| DLEU2 | hsa-miR-214 |
| DLEU2 | hsa-miR-761 |
| DLEU2 | hsa-miR-3619-5p |
| DLEU2 | hsa-miR-216a |
| DLEU2 | hsa-miR-216b |
| DLEU2 | hsa-miR-216b-5p |
| DLEU2 | hsa-miR-221 |
| DLEU2 | hsa-miR-222 |
| DLEU2 | hsa-miR-222ab |
| DLEU2 | hsa-miR-1928 |
| DLEU2 | hsa-miR-223 |
| DLEU2 | hsa-miR-122 |
| DLEU2 | hsa-miR-122a |
| DLEU2 | hsa-miR-1352 |
| DLEU2 | hsa-miR-23abc |
| DLEU2 | hsa-miR-23b-3p |
| DLEU2 | hsa-miR-25 |
| DLEU2 | hsa-miR-32 |
| DLEU2 | hsa-miR-92abc |
| DLEU2 | hsa-miR-363 |
| DLEU2 | hsa-miR-363-3p |
| DLEU2 | hsa-miR-367 |
| DLEU2 | hsa-miR-101 |
| DLEU2 | hsa-miR-101ab |
| DLEU2 | hsa-miR-30abcdef |
| DLEU2 | hsa-miR-30abe-5p |
| DLEU2 | hsa-miR-384-5p |
| DLEU2 | hsa-miR-124 |
| DLEU2 | hsa-miR-124ab |
| DLEU2 | hsa-miR-506 |
| DLEU2 | hsa-miR-375 |
| DLEU2 | hsa-miR-128 |
| DLEU2 | hsa-miR-128ab |
| DLEU2 | hsa-miR-129-5p |
| DLEU2 | hsa-miR-129ab-5p |
| DLEU2 | hsa-miR-499-5p |
| LINC00504 | hsa-miR-132 |
| LINC00504 | hsa-miR-212 |
| LINC00504 | hsa-miR-212-3p |
| LINC00504 | hsa-miR-140 |
| LINC00504 | hsa-miR-140-5p |
| LINC00504 | hsa-miR-876-3p |
| LINC00504 | hsa-miR-1244 |
| LINC00504 | hsa-miR-194 |
| LINC00504 | hsa-miR-196abc |
| LINC00504 | hsa-miR-205 |
| LINC00504 | hsa-miR-205ab |
| LINC00504 | hsa-miR-24 |
| LINC00504 | hsa-miR-24ab |
| LINC00504 | hsa-miR-24-3p |
| LINC00504 | hsa-miR-25 |
| LINC00504 | hsa-miR-32 |
| LINC00504 | hsa-miR-92abc |
| LINC00504 | hsa-miR-363 |
| LINC00504 | hsa-miR-363-3p |
| LINC00504 | hsa-miR-367 |
| LINC00504 | hsa-miR-124 |
| LINC00504 | hsa-miR-124ab |
| LINC00504 | hsa-miR-506 |
| LINC00504 | hsa-miR-338 |
| LINC00504 | hsa-miR-338-3p |
| LINC00504 | hsa-miR-375 |
| LINC00504 | hsa-miR-128 |
| LINC00504 | hsa-miR-128ab |
| LINC00504 | hsa-miR-129-5p |
| LINC00504 | hsa-miR-129ab-5p |
