# Supplementary figures and images for: Development of an immunogenomic landscape for the competing endogenous RNAs network of peri-implantitis
Source: BMC Med Genet. 2020 Oct 20;21:208. doi: 10.1186/s12881-020-01145-4 (PMC7576812; doi:10.1186/s12881-020-01145-4)

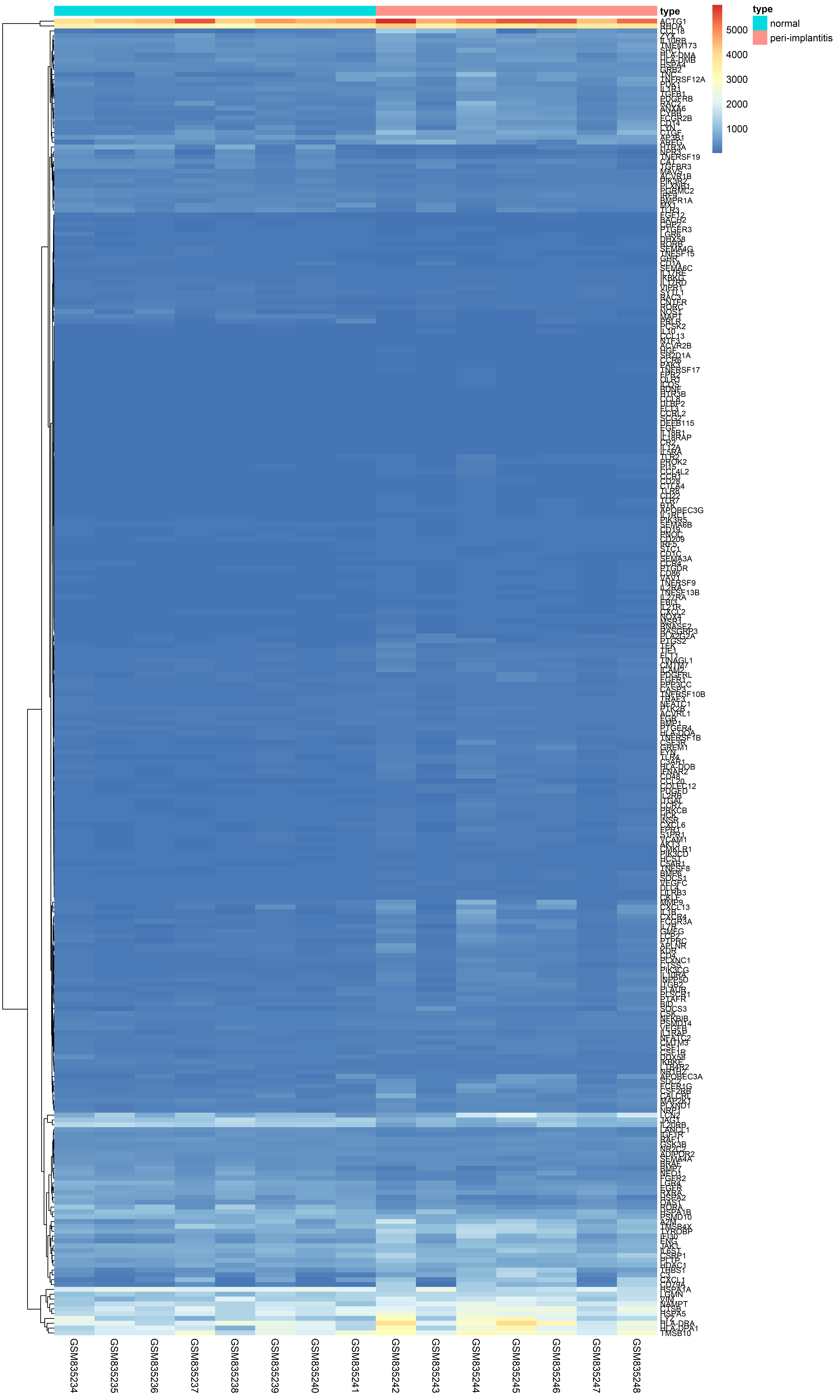

Supplement: Supplementary file 2 — Figure S1. Differentially expressed immune-related genes (IRGs) between peri-implantitis and normal tissues. (TIFF 2575 kb) [file 12881_2020_1145_MOESM2_ESM.tiff]
